# Gut Microbiota as Early Predictor of Infectious Complications before Cardiac Surgery: A Prospective Pilot Study

**DOI:** 10.3390/jpm11111113

**Published:** 2021-10-29

**Authors:** Ekaterina Chernevskaya, Evgenii Zuev, Vera Odintsova, Anastasiia Meglei, Natalia Beloborodova

**Affiliations:** 1 Federal Research and Clinical Center of Intensive Care Medicine and Rehabilitology, 25-2 Petrovka Str., 107031 Moscow, Russia; zuev17ev@gmail.com (E.Z.); amegley@fnkcrr.ru (A.M.); nvbeloborodova@yandex.ru (N.B.); 2 N. Pirogov National Medical Surgical Center, 70 Nizhnyaya Pervomayskaya Str., 105203 Moscow, Russia; 3 Atlas Biomed Group—Knomics LLC, 31 Malaya Nikitskaya Str., 121069 Moscow, Russia; odintsova@atlas.ru

**Keywords:** cardiovascular diseases, 16S RNA sequencing, microbiome, biomarkers, critically ill

## Abstract

Cardiac surgery remains a field of medicine with a high percentage of postoperative complications, including infectious ones. Modern data indicate a close relationship of infectious disorders with pathological changes in the composition of the gut microbiome; however, the extent of such changes in cardiac surgery patients is not fully clarified. In this prospective, observational, single center, pilot study, 72 patients were included, 12 among them with the infectious complications. We analyzed the features of the fecal microbiota before and in the early postoperative period, as one of the markers for predicting the occurrence of bacterial infection. We also discovered the significant change in microbial composition in the group of patients with infectious complications compared to the non-infectious group before and after cardiac surgery, despite the intra-individual variation in composition of gut microbiome. Our study demonstrated that the group of patients that had a bacterial infection in the early postoperative period already had an altered microbial composition even before the surgery. Further studies will evaluate the clinical significance of the identified proportions of individual taxa of the intestinal microbiota and consider the microbiota as a novel target for reducing the risk of infectious complications.

## 1. Introduction

Infections are frequent complications of cardiac surgery [1]. Despite the success in the development of surgical practices, this percentage does not decrease, and ranges from 12.6 to 21%. Among them, a significant portion is postoperative pneumonia and surgical site infections [2,3,4,5]. Risk factors include age, chronic lung disease, heart failure, duration of surgery, cardiopulmonary bypass, and others. The state of the microbiota is not taken into the account, although it may be a therapeutic target [6]. Metabolism-dependent and -independent processes are the link between the «gut–heart axis». On the one hand, the gut microbiota acts as an endocrine organ producing bioactive metabolites, including Trimethylamine/trimethylamine N-oxide (TMAO), short chain fatty acids and others. On the other hand, impaired cardiac activity contributes to bowel wall edema, resulting in bacterial translocation with the subsequent pro-inflammatory condition, which also impaired heart function [7,8]. The growing scientific evidence supports the role of the gut microbiota in the pathogenesis of heart failure [9,10,11,12,13,14]. However, only a very few studies are devoted to the composition of the gut microbiota of elective surgery patients in the intensive care unit [15]. 

Several studies of gut microbiota dynamics in ICU patients using 16S rRNA gene sequencing indicate a rapid disruption of gut microbiota during ICU stay, and this is associated with a loss of diversity and overgrowth of potentially pathogenic microorganisms [16,17,18,19]. The microbial disbalance in the gut may have a clinical relevance and can lead to inflammation and infection [20], also playing a potential role in neurological deficits [21].

More common in cardiac surgery, research is aimed at clarifying and early detection of compromised patients in order to provide customized management strategies that match the patient’s molecular and biochemical profile. Along with traditional biomarkers, NT-proBNP, hight-sensitive Troponine T, the use of novel biomarkers, such as microRNAs, mitochondrial peptides, inflammatory cytokines and adhesion molecules are discussed [22].

The aim of the study was to identify the features of changes in the gut microbiota and biomarkers in patients after cardiac surgery and to assess their relationship with postoperative complications. We observed that, despite the large interindividual variability of the microbial composition, the composition of the gut microbiota in patients with infectious complications showed a consistent pattern with the relative predominance of potentially pathogenic species.

## 2. Materials and Methods

### 2.1. Study Design

This prospective observational pilot study was performed in the N. Pirogov National Medical Surgical Center, Moscow, Russian Federation. The local Ethics Committee approved the study (no. 04 22.05.2018), which was conducted in accordance with the ethical standards of the Declaration of Helsinki. A formal consent for participation in this study was also obtained from each patient or his/her legal representative.

### 2.2. Patients and Samples

All patients have received perioperative antibiotic prophylaxis: cefazolin 3 times within 24 h for CABG or vancomycin 4 times within 48 h for valve surgery. The inclusion criteria are as follows: age over 18 years old, planned surgical intervention, patients with the following types of cardiac surgery—heart valve surgery, off-Pump CABG, CABG (Coronary artery bypass grafting), combination surgery—and signed informed consent to participate in the study. Exclusion criteria are as follows: active infectious endocarditis, emergency surgery, previous bacterial infectious diseases in the last three months, antibiotic intake in the last three months, inflammatory bowel disease, and patients refusing to participate in the study.

All stool samples and venous blood samples were collected from each patient before and after 1, 3 and 7 days of cardiac surgery. All samples before surgery were collected prior starting antibiotic prophylaxis. Blood was collected from a venous catheter into an anticoagulant-free test tube. Serum samples were obtained by centrifuging the blood at 1500× *g* for 10 min. Serum aliquots (500 μL) were poured into disposable Eppendorf tubes, frozen and stored at −20 °C until further use. Stool samples were obtained by collecting a small amount of feces as a rectal swab and dissolving it in 1 mL of sterile saline solution; after thorough mixing, it was divided into two Eppendorf tubes and were frozen and stored at −30 °C prior to analysis. 

### 2.3. Analysis of Serum Biomarkers

Neurological (S100), inflammatory (IL6), cardio (NT-proBNP, hight-sensitive Troponine T (hs-TnT)), “stress” (ACTH, cortisol) and bacterial infection (procalcitonin (PCT)) biomarkers were measured in 200 μL serum samples using reagent kits on automated electrochemiluminescence analyzer Cobas e411 (Roche; Basel, Switzerland). Biomarkers of gut microbiota metabolic activity (Taurine, Trimethylamine N-oxide (TMAO)) were measured using reagent kits by Cloud Clone, Katy, USA, on automated microplate photometer Multiscan (Thermo Scientific; Waltham, MA, USA) which relies on the linked immunosorbent assay.

### 2.4. Microbiome Sample Preparation

Defrozen fecal solution (200 mL) was placed in the 2.0 mL tube containing 3:1 mix of 0.1 mm and 0.5 mm pre-sterilized glass beads (Sigma, St. Louis, MO, USA). Then 1 mL of a warm 60 °C lysis buffer (500 mM NaCl, 50 mM Tris-HCl, pH 8.0, 50 mM EDTA, 4% SDS) was added. The mixture was vortexed and homogenized with MiniLys (Bertin Technologies S.A.S., Montigny Le Bretonneux, France) for 3 min. The lysate was incubated at 70 °C for 15 min and centrifuged for 20 min at 14,000 rpm. The supernatant (1 mL) was transferred to the sterile tube and put on the ice. The pellet was added to a 1 mL of lysis buffer and the homogenization process was repeated. The supernatants were combined in the 15 mL tubes with the addition of 4 mL of 96% ethanol and 200 μL of 3 M sodium acetate. The mixture was incubated at −20 °C for not less than 1 h. Then the mixture was centrifuged for 15 min at 14,000 rpm at +4 °C, the supernatant was discarded, the DNA pellet was washed twice with 80% ethanol. The pellet was dried at 53 °C for 30–60 min and resuspended in 200 μL of sterilized milliQ water. The mixture was centrifuged and transferred into new tubes. Resulting DNA solution was treated with 10 μL of RNAse A (5 mg/mL) for 1 h at 37 °C, followed by an additional round of chloroform purification. Chloroform was added to the solution in 1:1 ratio, tube was vortexed for 1 min and centrifuged at 5000× *g* for 5 min. Aqueous phase was transferred to new sterile tube and used for PCR dilutions. The obtained DNA solution was stored at −20 °C. Amplicon sequencing of V4 variable region of microbial 16S rRNA gene was performed on a MiSeq sequencer (Illumina, San Diego, CA, USA) as described before [23].

### 2.5. Microbiome Data Processing

Raw microbiome data is available in the Sequence Read Archive (SRA) by the accession number PRJNA688839. The reads were processed using the Knomics-Biota system [23,24] (“16S dada2 Greengenes V4”) pipeline based on the DADA2 algorithm and Greengenes database [25,26] as previously described [27]. In the pipeline, the Greengenes database was preprocessed using TaxMan [27,28] based on the F515-R806 primers for V4 region of the 16S rRNA gene. The sequences were clustered with 97% identity using cd-hit software version 4.8.1 [27,28,29]. The slash (“/”) character was used (example: (Blautia/Dorea)), for ambiguous sequences, for which taxonomy could not be resolved based on the used primers. When the sequence could not be resolved at a particular taxonomic rank, the “_u” sign was used (referring to the term “unclassified”, example: “Lactobacillus_u”). There were minor changes to the original Knomics-Biota pipeline: the Chao1 index calculated on the level of ASVs (amplicon sequencing variants) after rarefaction to 3000 reads per sample was used to assess the alpha diversity; the beta diversity was estimated using Euclidean distance in Aitchison space [30,31].

### 2.6. Statistical Analysis

This statistical analysis of microbiome composition was done in R [32]. Only genera that were presented by more than 10 counts in ≥50% of samples were included in the analysis (in total 40 genera). The abundance table was obtained with cmultRepl function from zCompositions library [33,34]. The Aitchison distance was used to estimate beta diversity between samples. Comparison of general proportions in two groups of samples was done in the following way: the independent balances containing 2 or 3 taxa were obtained with DBA-distal method [33], the statistical significance of association between these balances and infectious complications was assessed by linear regression analysis. The Benjamini-Hochberg correction was used to adjust for multiple testing. The adonis function from the vegan library was used for beta-diversity analysis [35]. Changes of microbiome in time were treated separately for subjects with and without complications. Time points were compared pairwise. For each pair of time points, all patients that provided samples in these two days were included in the analysis; patients that did not provide a sample in any of these timepoints were excluded. The analysis of taxa proportions and beta diversity was performed in a similar way. It was done for comparison of samples collected before the surgery, with several changes: subject identifier was included as the “strata” parameter in the adonis function, and as a random effect in a mixed effect model in balances analysis (instead of the linear regression). The lmerTest library was used to fit mixed effect models and estimate significance of its coefficients [36]. The analysis of association between microbiome composition and blood parameters was performed with selbal [37] for the samples collected before the surgery. Only associations that were repeated in at least 50% of iteration of the cross-validation procedure are presented in the Results section. Association of beta-diversity between samples with the difference in blood parameters was analyzed with adonis.

## 3. Results

### 3.1. Patients Characteristics

**Figure 1:**
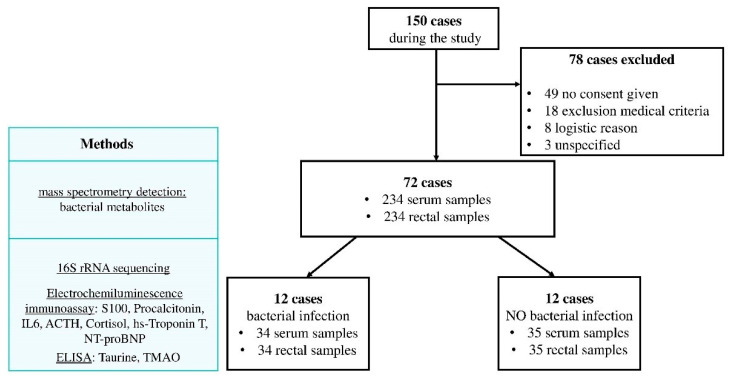
The study design. Of the 72 patients included in the study, complications developed in 12 cases. For comparison, a group of patients without complications was selected, comparable in the number of patients, age, severity of surgery and duration of the extracorporeal circulation.

The inclusion of patients and sample collections are shown in Figure 1. Of the 72 patients included in the study, complications developed in 12 cases. For further analysis of the gut microbiota and biomarkers, a group of patients without infectious complications was selected, comparable to infectious group.

Patients in both groups were comparable in terms of baseline, risks of surgery, age and duration of the extracorporeal circulation (Table 1).

We revealed significant differences between infectious and non-infectious groups on the SOFA scale and in lactate level on the first day after surgery, on the SOFA scale and WBC—on the 3rd and 7th day after surgery.

### 3.2. The Microbiota Composition

**Figure 2:**
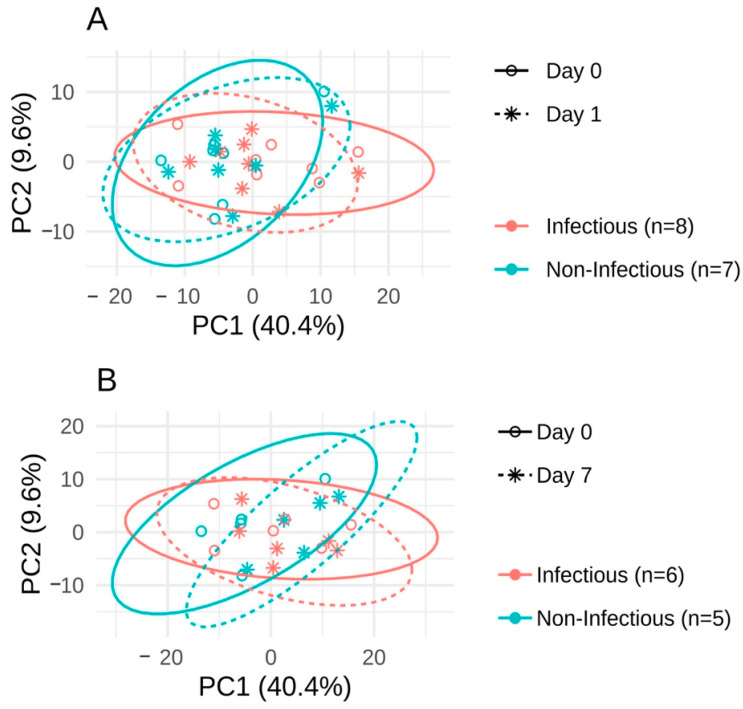
The principal component analysis PCA in ILR-coordinates for comparison of changes between: (**A**) the day before the surgery and the 1st day after it; (**B**) the day before the surgery and the 7th day after it. The principal component analysis was performed using all samples; coordinates on plots A and B are the same.

As a first step, we looked for microbiome predictors for the complications. The data obtained by 16S rRNA gene sequencing is known to be compositional [38], which means that ratios between taxa abundances, rather than themselves, should be explored. We used the Aitchison distance to measure the beta-diversity, and the DBA-distal method combined with linear regression analysis to find balances between groups of taxa that differed between subjects with and without complications before the surgery. Analysis of beta-diversity did not show significant difference in microbiome composition between infectious and non-infectious groups (adonis, *p* = 0.295, Figure 2).

**Figure 3:**
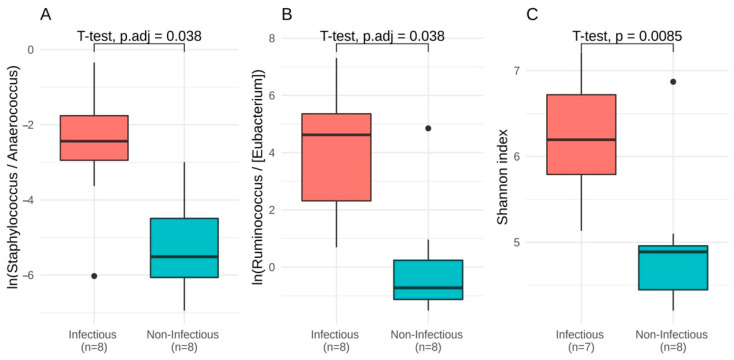
Difference between groups at the baseline. (**A**,**B**) Balances between taxa obtained by discriminative balance analysis and significantly different between groups. (**C**) Shannon index (only samples with total number of reads ≥ 3000 are included).

The groups differed in individual taxa proportions: the log-ratio of *Staphylococcus* to *Anaerococcus* and *Ruminococcus* to [*Eubacterium*] (*p* = 0.038 for each ratio) and Shannon index were higher in the infectious group (Figure 3) (*p* = 0.009, Welch test).

Beta-diversity analysis shows that for patients without infectious complications, samples (collected in the same time points) were not more similar than samples collected in different time points (adonis, *p* = 0.188). For the subjects with complications, they were more similar (*p* = 0.003). The post hoc pairwise comparison of time points showed that the most significant changes were observed in the period after the surgery (between 3 and 7 days, *p* = 0.03125). Changes (beta-diversity) in case and control groups did not differ significantly. The Shannon index did not change significantly in any of the groups.

Changes in individual taxa proportions are summarized in Table 2. All balances in Table 2 increased with time. An increase in the balance may indicate (1) an increase in the number of bacteria in the numerator, (2) a decrease in the number of bacteria in the denominator, or (3) an increase in the number of bacteria in both the numerator and the denominator, but much more in the numerator.

**Figure 4:**
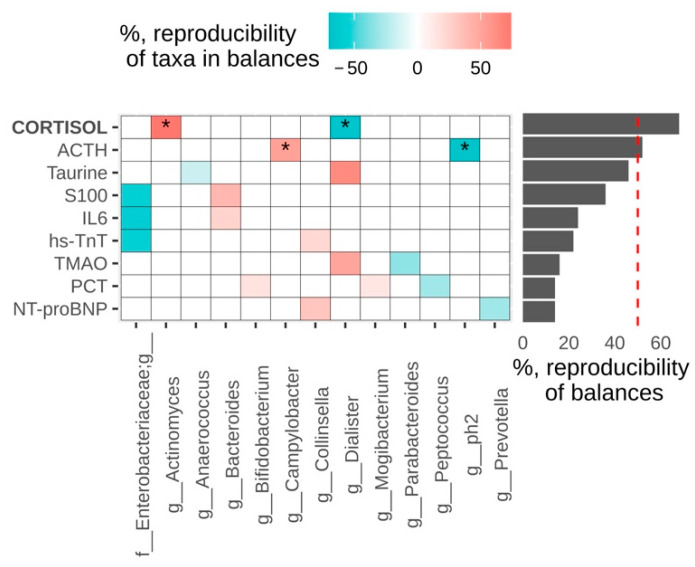
Associations between metabolites in blood and microbiome composition of samples collected day before the surgery. Reproducibility of balances and their components is measured as the proportion of cross-validation iterations in which they were observed. The balances with reproducibility higher than 50% are considered as reproducible; they are signed by *.

We also analyzed association of microbiome composition with biomarkers before the surgery (Figure 4).

Higher cortisol values were associated with increased log-ratio of *Actinomyces* to *Dialister*, higher ACTH—with higher *Campylobacter* to *ph2* log-ratio (genus from family [*Tissierellaceae*]). Significant associations between Aitchison distance and blood parameters were found for cortisol and TMAO (*p* = 0.044 for each of them).

### 3.3. Biomarkers

The study carried out a comparative assessment of the level of some laboratory markers between groups of patients with infectious complications and without infectious complications (Table 1A).

#### 3.3.1. Pro-BNP, HS-Troponin T Levels

**Figure 5:**
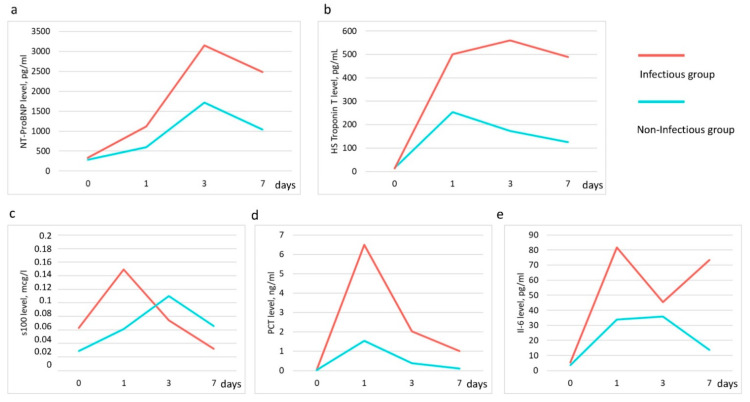
Dynamics of biomarker levels in patients’ groups: (**a**)—NT-ProBNP, (**b**)—high-sensetive Troponin T, (**c**)—protein S100, (**d**)-procalcitonin (PCT) and (**e**)—interleukin-6 (IL6).

Cardiac function was assessed by measuring NT-proBNP and hs-TnT. ProBNP and hs-TnT levels were higher in the group of patients with infectious complications compared to the group without infectious complications, but statistically significant differences between the two groups were found only for proBNP (*p* = 0.024). The pro-BNP level reached its maximum values three days after surgery in both groups (Figure 5a,b). 

#### 3.3.2. S100 Level

The degree of involvement in the pathological process of the central nervous system was assessed by the level of S100 in plasma. The maximum values were found on day 1 after surgery and amounted to 0.149 μg/L, with a gradual decrease in the following days (Figure 5c). The S100 level was higher in the infectious group. Clinical neurological features were observed in only four patients and were manifested by short-term delirium. There were no other signs of CNS damage. No association was found between delirium and change in S100 level.

#### 3.3.3. Interleukin—6 (IL), Procalcitonin (PCT) Level

As part of the assessment of the level of inflammation, PCT and IL-6 were investigated. In the group of patients with infectious complications, PCT levels were statistically significantly higher than in patients without infection (Figure 5d). The maximum values were observed on day one after surgery and reached 6.5 ng/mL and 1.54 ng/mL, with a further decrease to 1 ng/mL and 0.09 ng/mL in the infectious and non-infectious groups, respectively. IL-6 levels were also higher in the infectious group, but with no statistical difference (Figure 5e). The IL-6 level in both groups before surgery did not exceed the reference values (no more than 7 pg/mL). The IL-6 level in the non-infectious group did not exceed 35 pg/mL and decreased to 13 pg/mL by the seventh day after surgery. The level of IL-6 in the infectious group remained high, especially on the first and seventh days after surgery, 81.8 pg/mL and 73.4 pg/mL, respectively. 

#### 3.3.4. Adrenocorticotropic Hormone (ACTH), Cortisol Level

**Figure 6:**
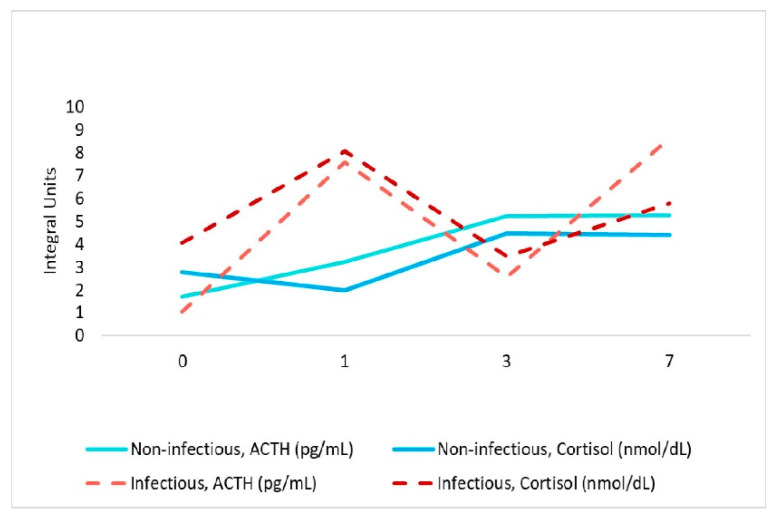
Association between ACTH and cortisol levels in dynamics in patients’ groups.

Stress levels were studied by assessing Cortisol and ACTH levels. Values of Cortisol exceeding the norm were found only in the group of patients with infectious complications on the first day after surgery and amounted to 806.1 nmol/L. They were statistically significantly different from the group of patients with non-infectious complication, median value 197.3 nmol/L at the first day after surgery (Figure 6). 

ACTH levels were within the normal range in both groups. In the group with infectious complications, the median values were lower than in the group with non-infectious complications.

#### 3.3.5. Taurine, TMAO Level

**Figure 7:**
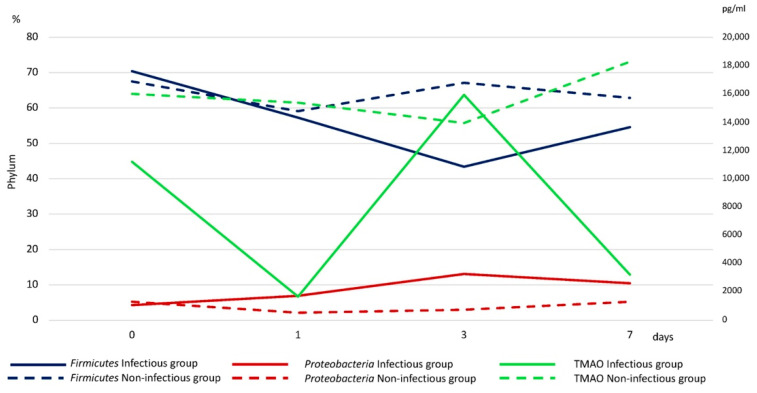
Association between metabolic activity of the gut microbiota by TMAO level (green line) and Phyla (*Firmicutes*—dark blue line, *Proteobacteria*—red line). The group of infectious complications is shown by a solid line, and the group of non-infectious complications is shown by a dotted line.

The level of intestinal metabolic activity was studied by assessing the levels of Taurine and TMAO. The TMAO values were lower in the infectious group compared with the non-infectious group (Appendix A. Table A1). The level of TMAO in the infectious group decreased on the first and seventh days and reached a maximum on the third day. The TMAO level in the non-infectious group tended to decrease by day three and slightly increase by day seven (Figure 7). This dynamic is similar to the change in the level of Proteobacteria. 

We found a positive correlation between Proteobacteria and TMAO in the non-infectious group (r = −0.38, *p* < 0.05), and a negative correlation between Firmicutes and Proteobacteria in the infectious group (r = −0.46, *p* < 0.05). Taurine levels were not statistically significant between two groups; however, the level of taurine in the group of patients with infectious complications was higher compared to the non-infectious group.

### 3.4. Clinical Cases with Microbiological Confirmation of Infection

**Figure 8:**
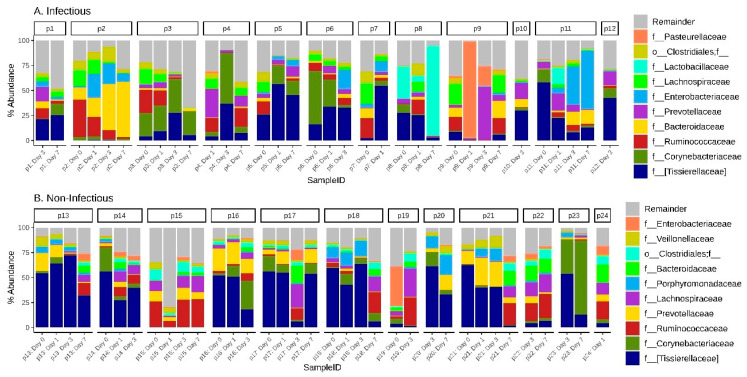
The top 10 families of the gut microbiota composition in both (**A**) infectious and (**B**) non-infectious groups of patients with temporal dynamics.

**Figure A1:**
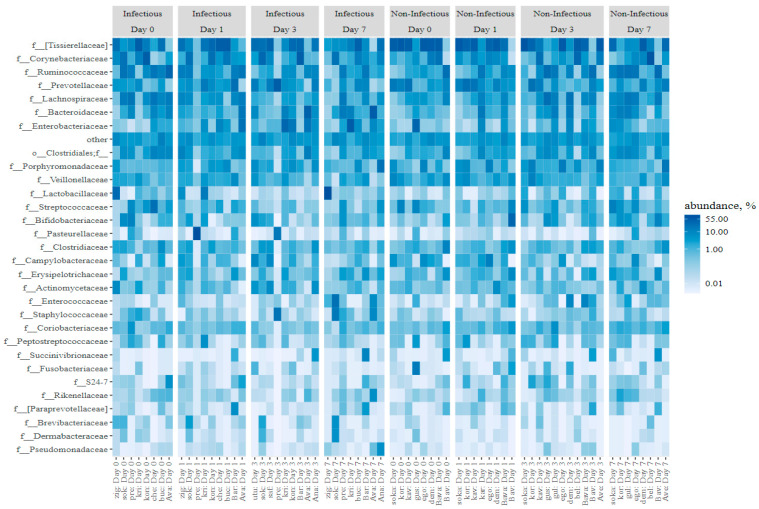
Heatmap of the temporal dynamics of the taxonomic composition of the intestinal microbiota in both infectious and non-infectious patient groups.

The dynamics of the gut microbiota composition in groups of patients is shown in Figure 8 and Appendix A. Figure A1. In five patients from the infectious group, microbiological confirmation was obtained with the identification of the pathogen. All these patients needed prolonged treatment with several classes of antibiotics and its correction, based on the obtained microbiological data. 

Patient 4 was admitted after CABG—early postoperative period without complications and with early extubation. He was discharged from the ICU on the second day. However, from the sixth day, leukocytosis, inflammation of the surgical wound (osteomyelitis) with the growth of Enterobacter cloacae, was revealed. In this patient, noticeable overgrowth of Corynebacteriaceae (52%) and [Tissierellaceae] (36.7%) was detected at d3. Anaerobic Gram-negative microorganisms predominated on day seven, belonging to the genera Succinivibrio and Prevotella (11.5 and 11.3, respectively), most of which are pathogenic species. The patient received successful antibiotic therapy and was then discharged home.

Patient 6 was admitted after CABG, extubated in the early hours. Acute postoperative myocardial infarction was developed on the second day. The patient was intubated due to pulmonary edema and unstable hemodynamics. From the third day, signs of lung infection appeared: low P/F, Xray picture, leukocytosis, increased PCT, with the growth of *Staphylococcus aureus*, *Serratia marcescens* and *Candida albicans* in BAL. In this patient, the relative abundance of *Corynebacteriaceae* was 53% at d0 decreasing to 3% by day 3, while at d3 the prevalence of [*Tissierellaceae*] (34%) and *Enterobacteriaceae* (19%) were detected. Clinical improvement was observed after resolution of heart failure and initiation of antibiotic therapy. 

Patient 8 was admitted after CABG on pump and suffered an intraoperative myocardial infarction and cardiogenic shock. The multiple organs failure progressed on the second day. Clinical signs of systemic infection were detected as confirmed by positive blood culture on day three with *Enterococcus faecalis*. The gut features are characterized by a relatively high abundance of *Lactobacillaceae* (25%) at d0 up to 90% to d7. The patient received massive antibiotic therapy and was transferred from the ICU in 12 days after stabilization. Length of hospital stay was 21 days.

Patient 9 was admitted after mitral valve replacement. Signs of organ failure were observed on the second day, which required prolonged mechanical ventilation. A picture of exacerbation of COPD, acute purulent bronchitis with the growth of *Haemophilus parainfluenzae biotype I*, *Paenibacillus lactis* and *Streptococcus salivarius* in BAL at d3. This patient, before and after surgery (d7), was prevalently closer to core of gut microbiota genera, such as *Lachnospiraceae*, *Ruminococcaceae*, *Bifidobacteriaceae*, *Bacteroidaceae*. At the same time, on the first day after the surgery, the number of *Pasteurellaceae* (including the genus *Haemophilus*) in the gut was 96%. The relative abundance of this family decreased to 19.5% in d3, with an increase of *Prevotellaceae* (54%) and *Staphylococcaceae* (23%). On the third day, antibacterial therapy was prescribed. Within seven days, the patient’s condition stabilized, after which he was discharged from the ICU.

Patient 11 also had nosocomial pneumonia with the growth of Pseudomonas aeruginosa in BAL at d6. The patient was admitted after CABG + aortic valve replacement. On the second day, multiple organ failure (kidneys, lungs, brain) was observed due to postoperative heart failure. Lung infection joined at 4–5 days. In this patient, noticeable overgrowth of [Tissierellaceae] (58%) and Corynebacteriaceae (14%) was detected at d0. The relative abundance of [Tissierellaceae] decreased, 22%, 12% and 8% at d1, d3, d7, respectively, with simultaneous growth of Enterobacteriaceae at d3 - 60% and 38% at d7. The patient spent 21 days in the ICU. His condition stabilized, then he was discharged from the hospital after 16 days.

## 4. Discussion

The high percentage of postoperative infectious complications in cardiac surgery poses a problem for the search for new markers that allow to identify high-risk patients. One of these markers may be the gut microbiota. In this prospective observational pilot study, we observed that, despite the large interindividual variability of the microbial composition, in patients with infectious complications, it characterized patterns with the relative predominance of potentially pathogenic species. 

One of the main findings of our study was that markers of infectious complications can be found in the proportions of individual taxa of the gut microbiota prior to surgery, in particular, by the log-ratio of the *Staphylococcus* to *Anaerococcus* and *Ruminococcus* to [*Eubacterium*]. Among them, *Staphylococcus* is the genus of facultative anaerobic bacteria which frequently colonizes the nares and skin in the healthy population, but in preoperative cardiac patients, carriage is associated with an elevated risk for post-operative surgical site infection and bacteremia [39,40]. *Anaerococcus* have the potential to metabolize peptones and amino acids and to produce short-chain fatty acids (SCFAs), such as butyric acid, but can be associated with skin and soft tissue infections and chronic wounds [41]. *Ruminococcus* and [*Eubacterium*] are usually the part of the resident microflora and also produce SCFAs; in some case, they may be players in the development of inflammation and bloodstream infection [42,43]. The balance on 1st day in infectious group is *Prevotella*/*Actinomyces*. Several members of [*Eubacterium*], *Actinomyces*, *Prevotella*, are anaerobic flora of the oral cavity and are the cause of infections, including purulent bacterial pericarditis [44]. Moreover, coaggregation was found between *Prevotella intermedia* strains and individual *Actinomyces* species via a protein or glycoprotein on the *Prevotella* strain, which can interact with carbohydrates or carbohydrate-containing molecules on the surface of the *Actinomyces* strain [45]. The predominance of representatives of the oral microbiota as part of the gut microbiota in the infectious group is highlighted by additional studies documenting the effect of patient participation in reducing the risk of postoperative infection by adhering to preoperative oral hygiene regimens [46].

**Figure A2:**
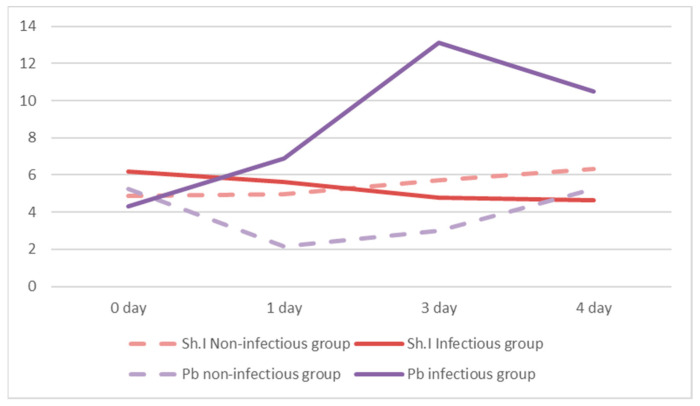
Association between Shannon Index (Sh.I) and Proteobacteria Phyla (Pb) in both Infectious and Non -infectious groups of patients with temporal dynamics. Decrease in Proteobacteria by 1 day in patients in the non-infectious group, most likely with surgical antimicrobial prophylaxis.

The Shannon Index was low in all patients, which was consistent with previously obtained data on its level, correlating with the heart failure class [47]. Nevertheless, before the surgery, the Shannon Index was higher in the group of infectious complications, but after the surgery this value decreased. While in patients without infectious complications, it increased. This phenomenon can be explained by an increase in the proportion of taxa from the *Proteobacteria* phylum in the patients with infection (Appendix A. Figure A2).

A review of studies showed that changes in taxonomic composition in the infectious group are consistent with earlier studies, where *Streptococcus*, *Blautia*, *Peptococcus*, *Porphyromonas* were associated with infective endocarditis, coronary heart disease, inflammation, sepsis, complications after stroke (Supplementary Table S1).

Assessing the metabolic activity of the microbiota, we compared of the results the study of taurine with the composition of the microbiota of patients using the 16S-sequencing method. This suggests that higher values of taurine in the infectious group may be associated with dysbiotic disorders, towards an increase in species metabolizing taurine, such as *Clostradiales* [48]. It is known that taurine decomposes to hydrogen sulphide under the influence of the intestinal microbiota. High concentrations of hydrogen sulfide can suppress the activity of cytochrome oxidases [49] and, consequently, aerobic respiration, one of the common factors of virulence of microorganisms [50]. Thus, taurine can be not only a nutrient for microbes; it can also stimulate the antimicrobial defense of the body [51] and have positive effects in cardiovascular diseases [52,53,54].

We found lower serum TMAO levels in patients with infectious complications compared to the group of patients without infectious complications. Serum TMAO levels are genetically determined and also depend on diet [55] and the composition of the intestinal microbiota [56]. Recent studies have reported that several families of bacteria belonging to the type *Firmicutes* and *Proteobacteria* are potential producers of TMA [57,58]. We compared the dynamics of changes in TMAO levels with the levels of *Proteobacteria* and Firmicutes in patients in both groups, using the ratio of *Proteobacteria* and *Firmicutes*. We found that increasing the ratio of *Proteobacteria* and *Firmicutes* associated with elevated levels of TMAO (Figure 7). Previous data indicate that this ratio was a predictor of adverse outcomes in cardiovascular disease [59].

At the same time, biomarker identification to personalize therapy in clinics is more common than microbiota research. Among them, markers of infectious, neurological and cardiac complications are distinguished. Inflammatory biomarkers IL6, PCT, cortisol, ACTH are not always specific for assessing the severity of the infectious process in cardiac surgery patients. Cardiac surgery causes an increase in the PCT level even in the absence of complications, and its level usually does not exceed 5 ng/mL [60,61]. At the same time, in the infectious group, the average value of serum PCT on the first day after surgery was 6.5 ng/mL, which is one of the reliable laboratory criteria for predicting the presence of a bacterial infection. IL-6 levels were also higher in the infectious group, but not statistical different. IL-6 is rarely used in the clinical practice of cardiac surgery due to its lower specificity than PCT [62]. ACTH levels were within the normal range in both groups. There is a direct relationship between an increase in cortisol levels and an increase in ACTH levels, which indicates a central regulation of the level of inflammation despite the administration of exogenous glucocorticosteroids (dexamethasone 80 mg) in high doses before surgery (Figure 6). Only four patients in this case had cortisol values below 149 nmol/L, which may be a sign of adrenal insufficiency, but all four either had no complications after surgery or had minimal complications that did not lengthen the number of days of treatment. Remarkably, among biomarkers and individual taxa, only for high cortisol values and ACTH were found significant association, with increased log-ratio of *Actinomyces* to *Dialister* and with high *Campylobacter* to *ph2* log-ratio (genus from family [*Tissierellaceae*]), respectively.

Endogenous intoxication due to infection can worsen the function of the heart, which can manifest itself both clinically and in a laboratory. In our study, we found higher NT-proBNP values in patients with infectious complications. (Figure 5) NT-proBNP is used in the diagnosis of heart failure. Its values always increase during the first few days after open heart surgery with a further gradual decrease if there are no complications. Typically, NT-proBNP values are higher in more severe patients receiving inotropic therapy. Another promising biomarker to evaluate postoperative complication is high-sensitive Troponin T levels, the release of which should be expected after all CABG procedures. It depends on the procedure, the nature of the cardioplegia and many other factors. According to the Fourth Universal Definition of Myocardial Infarction, CABG-related MI is arbitrarily defined as elevation of troponin values more 10 times the 99th percentile upper-reference limit in patients with normal baseline troponin values in combination with other objective signs of myocardial ischemia [63]. The peak hs-TnT usually occurs within 24 to 48 h after operation [64]. In this study, the level of troponin in the group of infectious complications was significantly higher than in the group of patients without infections. This can be explained by the fact that infectious complications developed more in those patients who had primary cardiac complications in the intraoperative or early postoperative period: myocardial infarction, myocardial injury, severe heart failure. Patients with primary cardiac complications have a greater risk of bacterial infection [65]. However, our findings correlate with data from the study that includes 1318 patients after CABG surgery, with a peak high-sensitivity troponin T level, greater than 400 ng/L measured within 24 h associated with a major adverse cardiac or cerebrovascular event, 30-day mortality, myocardial infection and ICU stay >48 h [66]. Prospectively designed trials may provide further insight into the prognostic value of high-sensitivity troponin T after cardiac surgery.

The S100 as an early marker for damage to the blood–brain barrier and neurons [67] could be used for a risk stratification of cardiac surgical patients for cognitive dysfunction [68] and postoperative delirium [69]. We did not reveal an association between delirium and S100 serum level; at the same time, this marker was higher in the infectious group on the first day after surgery followed by a decline. Studies have shown that the level of serum S100 protein in bacterial infection is significantly higher than that in viral infection [70,71], so it can also be used in combination with other markers to predict infectious complications.

This study has several limitations: a relatively small sample size, due to the impossibility of obtaining stool samples; the widespread use of antibiotics in different combinations in patients with infectious complications and intraoperative complications, which could significantly affect the composition of the microbiota and biomarker levels; and the inclusion multiple valve operations per study group. To exclude bias due to the inclusion of valvular surgeries, we additionally conducted a comparative analysis of patients with valvular surgeries and CABG with each other. Patient groups were similar in all parameters, except for antibiotic prophylaxis and blood loss, but the volume of blood loss during CABG was greater than during valvular procedures (1400 mL vs. 950 mL, respectively, *p* = 0.04). For this reason, the addition of valve surgery in this case is not a significant factor influencing the outcome and the possibility to develop infection, despite the fact that some valve operations are more difficult and time consuming. However, despite these limitations, our project makes it possible to assess the contribution of the taxonomic composition of the microbiota before and in the first days after surgery in dynamics.

## 5. Conclusions

The adaptation of treatment to the individual characteristics of each patient is the goal of personalized medicine. Clinical signs are faster but are nonspecific tools for this. Specific biomarkers are rapid and make it possible to identify groups of patients compromised with various types of complications. The gut microbiota is a major contributor to the pathophysiological process and may be a potential early biomarker. Predictably, patients with cardiovascular diseases have pronounced imbalances in the taxonomic composition of their gut microbiota. However, even before surgery, markers of subsequent infectious complications can be identified.

Further research is needed to confirm the role of the gut microbiota in the pathogenesis of development of infectious complications in surgical patients. Potentially, microbiota-targeted therapies could significantly improve the effectiveness of cardiac surgery.

## Figures and Tables

**Table 1:** Patients’ baseline characteristics.

Characteristic	Infectious Group	Non-Infectious Group	*p*
Age	66 (63; 71)	65 (62; 68)	0.378
Ejection Fraction	58 (43; 64)	60 (50; 66)	0.630
EuroScore 2	1.2 (0.7; 1.6)	0.9(0.7; 1.27)	0.318
The total duration of the extracorporeal circulation	90 (83; 107)	85 (66; 138)	0.630
1st day			
WBC (at the end of the 1st day)	17.3 (14.3; 24.8)	14.6 (11.7; 16.3)	0.178
Lactate max, during the 1st day, including EC	7.4 (3.5; 9.4)	4.5 (2.7; 5.9)	0.03
SOFA	6 (5; 9)	5 (2; 6)	0.03
3rd day			
WBC (at the end of the 3rd day)	16.5 (13.3; 22.5)	10.5 (8.7; 13.8)	0.01
SOFA	8 (6; 10)	1 (1; 3)	0.0001
7th day			
WBC (at the end of the 7st day)	11.9 (8.1; 16.3)	7.1 (5.9; 9.7)	0.01
SOFA	3 (1; 6)	0 (0; 0)	0.00001
Length of hospital stay, days	20 (15; 35)	13 (13; 14)	0.001

**Table 2:** Significant changes in individual taxa proportions after the surgery. The preliminary balances list was obtained by the discriminative balance analysis (DBA). The statistical significance of each balance change was tested by a linear mixed effect model with subject identifier as a random effect. Benjamini-Hochberg method was used to adjust for multiple testing.

		Day 1, Balance (p. adj)	Day 3, Balance (p. adj)	Day 7, Balance (p. adj)
Infectious				
	Day 0	*Prevotella*/*Actinomyces* (0.043)	*Porphyromonas*/*Streptococcus* (0.039) *u_Clostrideacea*/*Blautia* (0.039) *Bacteroides*/*Faecalibacterium* (0.039) *Corynebacterium*/*Peptococcus* (0.039) *Parabacteroides*/*u_Lachnospiraceae* (0.039)	*u_Lachnospiraceae*/[*Eubacterium*] (0.013) *Bacteroides*/*Ruminococcus* (0.013)
	Day 1	-	-	-
	Day 3	-	-	-
Non-Infectious				
	Day 0	-	*u_Lachnospiraceae*/*Faecalibacterium* (0.04) *Dorea*/[*Ruminococcus*] (0.032)	*Clostridium*/*Oscillospira* (0.049) *Bacteroides*/*u_Mogibacteriaceae* (0.049) [*Ruminococcus*]/*Dialister* (0.049)
	Day 1	-	*Lactobacillus*/*u_*[*Mogibacteriaceae*] (0.045) *Finegoldia*/*Peptoniphilus* (0.045) *Porphyromonas*/*Campylobacter* (0.045) *Faecalibacterium*/*Sutterella* (0.009)	*u_Clostridiaceae*/*Oscillospira* (0.029) *Bacteroides*/*u_*[*Mogibacteriaceae*] (0.04) *Collinsella*/*Dialister* (0.029) [*Ruminococcus*]/*Sutterella* (0.018)
	Day 3	-	-	-

## Data Availability

Raw microbiome data are available in the Sequence Read Archive (SRA) by the accession number PRJNA762260.

## Supplementary Materials

The following are available online at https://www.mdpi.com/article/10.3390/jpm11111113/s1, Table S1. The clinical significance of the found taxa. Data review.

## Appendix A

**Table A1:** Levels of biomarkers in groups of patients with infectious complications and without infectious complications. Data are presented as median and interquartile range (IQR).

Biomarkers	Patients (Median [IQR 25–75%])				
	Infectious Group		Non-Infectious Group		*p*-Level
ACTH, pg/mL	2.59	(1.26–8.7)	3.47	(1.4–7.3)	1
* Cortisol, nmol/L	473.2	(344.1–805.5)	384.25	(209.8–482.6)	0.037
IL-6, pg/mL	41.84	(7.7–83.03)	19.57	(7.04–37.7)	0.127
* PCT, ng/mL	0.924	(0.27–5.76)	0.183	(0.025–0.65)	0.002
* pro-BNP, pg/mL	1679	(674.25–4315.5)	908	(462.5–1378)	0.024
Troponin T-HS, pg/mL	279.4	(18.87–628.5)	126.9	(19.69–253)	0.369
S100, μg/L	0.084	(0.036–0.1375)	0.053	(0.031–0.136)	0.657
TMAO, pg/mL	9764	(1675–19,300)	15,848	(11,127–19,844)	0.052
Taurine, pg/mL	694	(379–769)	455	(325–720)	0.101

* Statistically significant differences.
