# Supplementary material for: Gut Microbiota as Early Predictor of Infectious Complications before Cardiac Surgery: A Prospective Pilot Study
Source: J Pers Med. 2021 Oct 29;11(11):1113. doi: 10.3390/jpm11111113 (PMC8622065; doi:10.3390/jpm11111113)
Supplement: Supplementary file 1 [file jpm-11-01113-s001.zip › jpm-1396525-supplementary.pdf]

Table S1. Review of the clinical significance of the taxa found.

| *Genus* | *Family* | *Order* | *Phylum* | Diseases |
|---|---|---|---|---|
| *Streptococcus* | *Streptococcaceae* | *Lactobaciales* | *Firmicutes* | mitral valve endocarditis (Nima Yaftian 2020) |
| | | | | tricuspid valve endocarditis (Zhiwei Xu 2017) |
| *Staphylococcus* | *Staphylococcaceae* | | | tricuspid valve endocarditis (Zhiwei Xu 2017) |
| *Peptococus* | *Peptococcaceae* | *Clostridiales* | | neointimal hyperplasia after arterial angioplasty (Cori A Cason 2020) |
| *Blautia* | *Lachnospiraceae* | | | coronary artery disease (Z. Liu et al., 2019) |
| | | | | post-stroke depression (Jiang et al., 2021) |
| | | | | Clostridioides difficile infection (Gu et al., 2020) |
| *Dorea* | | | | - |
| *Oscillospira* | *Ruminococcaceae* | | | heart failure (rats) (Gutiérrez-Calabrés et al., 2020) |
| | | | | PANS / PANDAS (Quagliariello et al., 2018) |
| | | | | Sepsis (mice) (L. Chen et al., 2019) |
| *Finegoldia* | *Peptoniphilacea* | | | Inflammation (Neumann et al., 2020) |
| | | | | infective endocarditis (Chien et al., 2019) |
| | | | | GPAC bacteraemia (Badri et al., 2019) |
| | | | | pacemaker pocket infection (Hosseini Dehkordi & Osorio, 2017) |
| | | | | |

| | | | | |
|---|---|---|---|---|
| | | | | Prosthetic Valve Endocarditis (Cobo et al., 2019) |
| *Peptoniphilus* | | | | Surgical site infection (Enault et al., 2020) |
| | *Mogibacteriaceae* | | | polytrauma hemorrhage model (rats) (Nicholson et al., 2018) |
| | | | | inflammatory bowel disease (Altomare et al., 2019) |
| | | | | intermittent hypoxia and hypercapnia mouse models of atherosclerosis-ApoE (Tripathi et al., 2019) |
| | | | | autoimmune diseases (Tripathi et al., 2019; Volkova & Ruggles, 2021) |
| *Dialister* | *Veillonellaceae* | *Veillonellales* | | Coronary heart disease (F. Liu et al., 2020; Tripathi et al., 2019; Volkova & Ruggles, 2021) |
| | | | | Early-onset neonatal sepsis (Dornelles et al., 2020) |
| | *Tissierellaceae* | *Tissierellales* | | inflamed and non-inflamed sites in patients with ulcerative colitis (Hirano et al., 2018) |
| *Sutterella* | *Sutterellaceae* | *Burkholderiales* | *Proteobacteria* | Cardiovascular Pathological Changes (Zhang et al., 2019) |
| | | | | Clostridioides difficile Colonization (Han et al., 2020) |

| | | | | |
|---|---|---|---|---|
| | | | | gastroenteritis and septicemia (mice) (Wang et al., 2020) |
| | | | | (Wang et al., 2020) |
| *Campylobacter* | *Campylobacteraceae* | *Campylobacterales* | | Campylobacter jejuni-associated perimyocarditis (Elford et al., 2021; Hessulf et al., 2016) |
| | | | | Campylobacter jejuni myocarditis (Obafemi et al., 2017) (Daboussi et al., 2020) |
